# Sarcopenia prevalence and associated factors among older Chinese population: Findings from the China Health and Retirement Longitudinal Study

**DOI:** 10.1371/journal.pone.0247617

**Published:** 2021-03-04

**Authors:** Xin Wu, Xue Li, Meihong Xu, Zhaofeng Zhang, Lixia He, Yong Li

**Affiliations:** 1 Department of Nutrition and Food Hygiene, School of Public Health, Peking University, Beijing, China; 2 Peking University Research Center on Aging, Beijing Key Laboratory of Protein Posttranslational Modifications and Cell Function, Department of Biochemistry and Molecular Biology, School of Basic Medical Science, Peking University, Beijing, China; 3 School of Public Health, Zhejiang University, Hangzhou, China; 4 Division of Molecular and Cellular Oncology, Dana-Farber Cancer Institute, Brigham and Women’s Hospital, Harvard Medical School, Boston, Massachusetts, United States of America; Ritsumeikan University, JAPAN

## Abstract

Sarcopenia a recognised geriatric syndrome. This study aims to evaluate the prevalence of possible sarcopenia, sarcopenia and severe sarcopenia among older Chinese adults and to identify any associated factors for possible sarcopenia according to the updated diagnostic criteria of the Asian Working Group for Sarcopenia 2019 (AWGS 2019). We used data from the China Health and Retirement Longitudinal Study (CHARLS). The main outcome of this study was possible sarcopenia. Handgrip strength was measured via a dynamometer. The muscle mass was estimated by anthropometric measures. Physical performance was measured by 5-time chair stand test and gait speed test. A multivariate logistic regression model with stepwise method was employed to identify factors associated with possible sarcopenia. A total of 6172 participants aged 60–94 years were included. The prevalence of possible sarcopenia, sarcopenia and severe sarcopenia was 38.5%, 18.6%, and 8.0%, respectively. Age, rural area, falls, higher C-reactive protein (CRP), and chronic diseases (including hypertension, chronic lung diseases, heart disease, psychiatric disease and arthritis) were associated with a higher risk of possible sarcopenia. Conversely, alcohol consumption, higher gait speed and high levels of hemoglobin were associated with decreased risk of possible sarcopenia. However, the associations between possible sarcopenia with alcohol consumption, heart disease, psychiatric disease and hemoglobin were not significant after Bonferroni correction. Our study reported a relatively high prevalence of sarcopenia among older Chinese population, and identified a range of factors associated with sarcopenia. We also found rural elders are more vulnerable to sarcopenia than urban elders. Additionally, we discovered systemic inflammation might be one of the contributing factors between sarcopenia and related comorbidities. We believe the findings of this study would help to identify individuals at high risk of sarcopenia early and therefore implement the prevention and treatment strategies to reduce the disease burden in China.

## Introduction

Sarcopenia, a progressive and generalized skeletal muscle disorder, is associated with an increased likelihood of adverse consequences such as falls, fractures, physical disability and mortality [1]. The presence of sarcopenia increases the risk for hospitalization and the costs of care during hospitalization [2]. It is well known that China is one of the fastest aging countries in the world. Sarcopenina will have a significant impact on Chinese people in the near future. Therefore, it is very important to know the prevalence of sarcopenia across China and identify the patients with possible sarcopenia and ulteriorly reduce the incidence of sarcopenia and severe sarcopenia in order to improve the health and quality of life for older people.

Some epidemiology studies from Asian countries which used the Asian Working Group for Sarcopenia 2014(AWGS 2014) criteria showed that the prevalence of sarcopenia was from 5.5% to 25.7% [3]. Currently, there are many studies carried out to investigate the prevalence of sarcopenia around the world. However, to present, there have not been large population-based perspective surveys on the national prevalence and associated factors of sarcopenia in China. Some studies demonstrated that aging, poor nutritional status, smoking and low BMI were risk factors for sarcopenia [4, 5]. Previous studies showed that the prevalence of sarcopenia increased in those with chronic obstructive pulmonary disease (COPD) [6], chronic heart failure [7] and chronic liver disease increased [8]. In addition, sarcopenia is associated with many other factors. The associated factors of sarcopenia in Chinese population may differ from other countries due to diverse genetic background, ethnicity, and living environment. Therefore, it’s vital to estimate the national prevalence of sarcopenia and identify the associated factors for Chinese population and develop specific prevention and treatment strategies for this disease in China.

Recently, the Asian Working Group for Sarcopenia 2019 consensus (AWGS 2019) redefined the sarcopenia including possible sarcopenia, sarcopenia and severe sarcopenia and cut-off points [3]. Therefore, the results from the former studies that applied the previous definition of sarcopenia may be no longer appropriate. It was the first time to recommend the concept of possible sarcopenia: the presence of low muscle strength with or without reduced physical performance, as a basis on which to begin intervention if assessment of muscle mass were not available [3]. Following the European Working Group on Sarcopenia in Older People 2 (EWGSOP2), muscle strength was recognized that was better than muscle mass in predicting adverse outcomes [1]. Therefore, we aimed to find factors associated with possible sarcopenia in Chinese population in order to increase awareness of sarcopenia and treat patients at risk for sarcopenia as early as possible.

## Methods

### Study population

CHARLS is a nationally representative investigation among the middle-aged and older population aged 45 years and older in China. CHARLS used multistep probability sampling strategy, selecting 150 representative counties and 450 villages/urban communities from 28 provinces. The detailed methodology description and core questionnaire of the CHARLS have been described in previous studies [9–11]. The national baseline survey for the CHARLS began in 2011, and the respondents were followed every two years by using a face-to-face computer-assisted personal interview. With the passage of time, loss to follow occurred, and the new respondents would be added. 17708 respondents were interviewed in 2011, 18605 respondents were interviewed in 2013, and 21095 were interviewed in 2015. The CHARLS was approved by the Ethical Review Committee of Peking University, and all participants signed informed consent at the time of participation.

**Fig 1:**
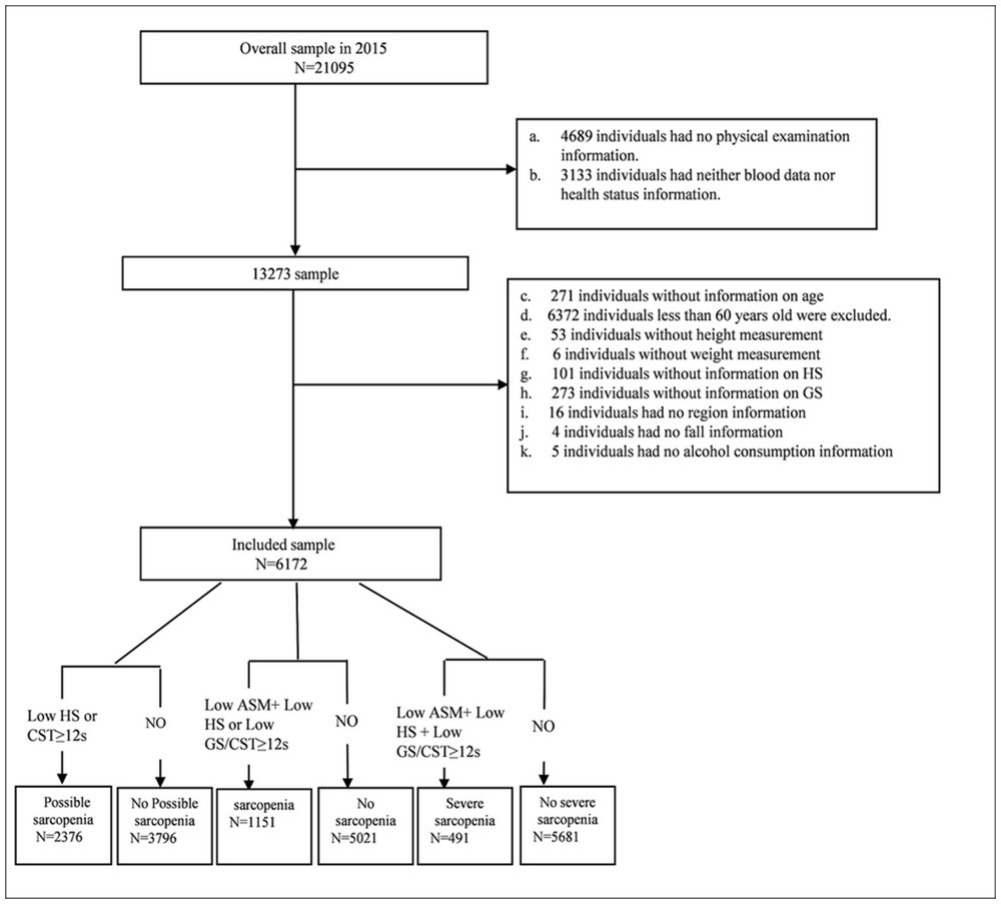
Flow of participants in the study. HS, handgrip strength; GS, gait speed; ASM, appendicular skeletal muscle mass, CST, chair stand text.

For our analysis, we used data from 2015 CHARLS. After excluding individuals aged less than 60 years and those with missing or invalid data on health status, blood data, physical examination and so on, 6172 participants aged 60 years and above were included in the present study. The detailed exclusion process was shown in the Fig 1.

### Assessment of possible sarcopenia, sarcopenia and severe sarcopenia

In this study, we adopted the recommended diagnostic algorithm of AWGS 2019. Possible sarcopenia is defined by low muscle strength with or without reduced physical performance. Sarcopenia is diagnosed when low muscle mass plus low muscle strength or low physical performance are detected. When low muscle strength, low muscle mass and low physical performance are all detected, severe sarcopenia will be considered [3]. The participants without any low muscle strength, low muscle mass and low physical performance were classified as ‘no sarcopenia’.

### Measurement of muscle strength

AWGS 2019 recommends using handgrip strength to indicate skeletal muscle strength [3]. Handgrip strength was measured by a trained examiner using a YuejianTM WL-1000 dynamometer (Nantong Yuejian Physical Measurement Instrument Co., Ltd., Nantong, China) in kilograms [9]. Subjects were in standing position and began the test using dominant or nondominant hand while receiving verbal encouragement. Every participant was measured twice for both left and right hand by holding the dynamometer at a right angle (90°) and squeezing the handle for a few seconds. Participants were asked to provide maximum effort for the measures [11]. We used the maximum available values measured twice with left and right hand, and then took the average of maximum available values. If the participants unable to perform grip strength measurement in either hand due to health reasons (swelling, inflammation, severe pain, or injury), we would use the value measured by the other hand. According to the suggestion of AWGS 2019, the cut-off points for low handgrip strength are <18 kg in women and <28 kg in men [3].

### Measurement of muscle mass

Height was measured using a stadiometer. Body weight was measured using a scale. The muscle mass was estimated by the appendicular skeletal muscle mass (ASM) using a previously validated anthropometric equation in a Chinese population [12]:

ASM=0.193*bodyweight+0.107*height–4.157*sex–0.037*age–2.631.

The height, body weight and age were measured in centimeters, kilograms and years, respectively. For sex, the value 1 represented men, and the value 2 represented women. The agreement of the ASM equation model and dual X-ray absorptiometry (DXA) was strong. The adjusted R^2^ of the equation model was 0.90, and the Standard Error of Estimate was 1.63 kg [12, 13]. After calculating the ASM, the height-adjusted muscle mass (ASM/Ht^2^) was calculated using the ASM divided by the square of the height in meters. Similar to previous studies [14–16], the cut-off for defining low muscle mass was based on the ASM/Ht^2^ of the lowest 20th% percentile of the study population. Therefore, the ASM/Ht^2^ values of <5.08 kg/m^2^ in women and <6.88 kg/m^2^ in men are defined as low muscle mass.

### Measurement of physical performance

Gait speed was assessed by measuring the participants’ usual gait (in m/s) in a 2.5m course. According to the requirement, the participants walked a 2.5m course at their normal pace. They walked the course at their usual pace twice (there and back). A stopwatch was used to time how fast the participant could walk [17]. We took the average of available values twice.

The chair stand text measures the amount of time needed for the participants to rise continuously five times keeping their arms folded across their chest from a chair. We used the value of amount of time participants held stand in seconds. We considered those who tried but failed to perform the chair stand text as low physical performance for the purpose of analyses.

According to AWGS 2019, the criteria for low physical performance are 6-m walk <1.0 m/s, or 5-time chair stand test ≥12 seconds, or Short Physical Performance Battery score (SPPB) <9 [3].

### Demographic characteristics

Demographic characteristics included age, sex, and residence. Age and sex was identified by their ID cards. Age was divided into three groups at every 10 years: 60–69, 70–79, and older than 80. The area type of their residence was categorized as “urban” or “rural” according to National Bureau of Statistics. An urban area is located in the city or city suburb, town or town suburb, or other non-agricultural industries, and accounted for>70% of the special areas such as special economic zone, state-owned agricultural enterprises, etc. The other area is the rural area [10].

### Health status

Body mass index (BMI) is defined as weight divided by height squared. BMI was divided into three categories according to the following WHO cut-off points for Chinese: underweight (BMI < 18.5 kg/m^2^), normal weight (BMI = 18.5 kg/m^2^ to 24 kg/m^2^), and overweight or obese (BMI ≥ 24 kg/m^2^) [18, 19].

Information on health conditions was based on doctors’ diagnosis of self-reports. Respondents were asked if they had been diagnosed with conditions listed below by a doctor (yes or no): hypertension, dyslipidemia, diabetes, cancer (excluding minor skin cancers), chronic lung diseases, liver diseases (except for fatty liver), heart diseases (heart attack, coronary heart disease, angina, congestive heart failure, or other heart problems), stroke, kidney disease, stomach or other digestive diseases, psychiatric problems, memory-related diseases, arthritis and asthma. Information on fall, fracture, smoking, drinking were based on self-reports. Fall and fracture (“Have you fallen down/ fractured your hip? or Have you fallen down/ fractured your hip since the last interview?” yes or no). Smoking status (“Have you ever chewed tobacco, smoked a pipe, smoked self-rolled cigarettes, or smoked cigarettes/cigars? The answer was “yes” or “no.”). Alcohol consumption was classified into non-drinker and drinker.

### CRP and hemoglobin measures

Venous blood samples were took to the Centers for Disease Control and Prevention (CDC) station, then immediately stored frozen at −20°C, and transported to the Chinese CDC within two weeks where they were placed in a deep freezer and stored at −80°C until the relevant assay was performed. The detection method of C-reactive protein (CRP) was immunoturbidimetric assay. Hemoglobin was measured at local county health centers.

### Statistical analyses

Statistical analyses were performed using IBM SPSS Statistic version 18.0 (IBM, Armonk, NY, USA). The continuous data were presented as the mean ± Standard Deviation (SD) if they were normally distributed. Absolute numbers and percentages were used to present the categorical variables. In order to compare the differences between groups, Student’s t-test or Mann-Whitney U test was used to compare the differences for continuous variables, whereas the Pearson chi-squared test was used for categorical data. A *p*-value less than 0.05 were considered statistically significant. The associated factors of possible sarcopenia was estimated by odds ratio (OR) and 95% confidence intervals (CI) from multivariate logistic regression. Variables with p ≤ 0.10 from the univariate analysis (t-test, Pearson chi-squared test) were entered into the multivariate logistic regression model. The logistic regression model was constructed with stepwise and forward elimination algorithms (forward: LR) used to identify the independently associated factors of possible sarcopenia. Goodness-of-fit for multivariate logistic regression models was assessed using the Hosmer-Lemeshow test (H-L test). Bonferroni correction was used to estimate the *p*-value for the multiple logistic model, to counteract the effect of multiple testing. Correlations between variables were analyzed using Pearson’s correlation coefficient. We also performed subgroup analyses according to residence area.

## Result

### The prevalence and characteristics of participants with possible sarcopenia

**Fig 2:**
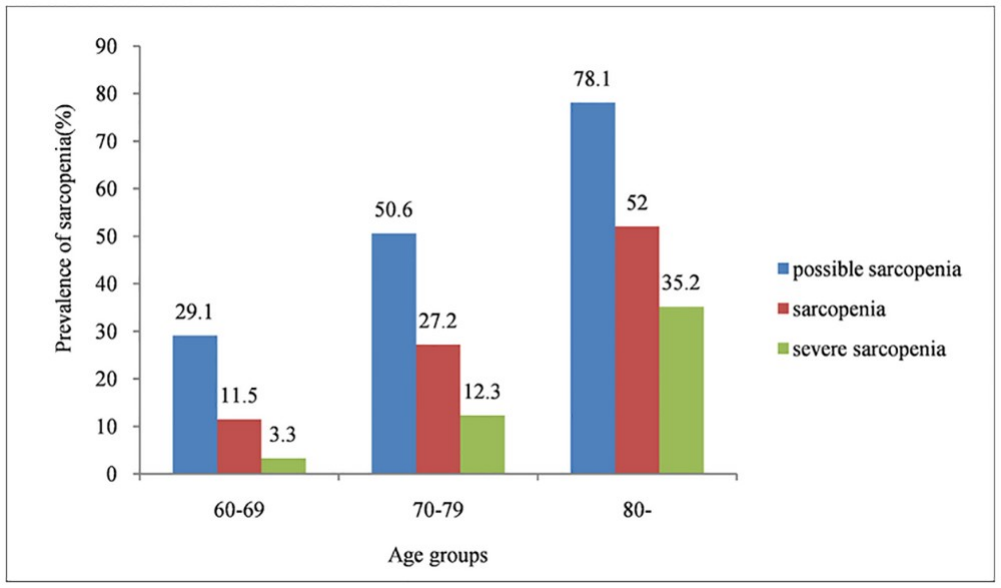
Prevalence of three categories of sarcopenia in different age groups.

**Fig 3:**
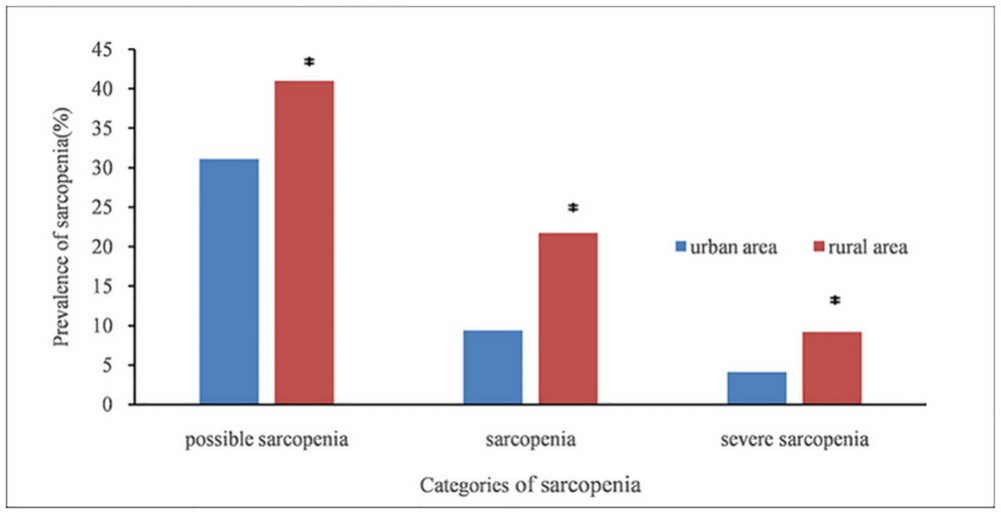
Prevalence of three categories of sarcopenia in urban and rural areas. * *p*<0.001.

Fig 1 shows the results according to the suggested diagnostic algorithm of AWGS 2019. A total of 6172 participants aged 68.13±6.46 years (range, 60–94 years) were included in the present study. As shown in Table 1 and Fig 1, possible sarcopenia had an overall prevalence of 38.5% (95% CI 37.3, 39.7). The prevalence of possible sarcopenia was 36.3% (95% CI 34.6, 38.0) among men and 40.7% (95% CI 38.9, 42.4) among women (*p* = 0.001). As shown in Fig 2, possible sarcopenia became more common with the aging: 29.1%, 50.6%, and 78.1% in the 60–69, 70–79, and ≥80 years groups, respectively. As shown in Table 1 and Fig 3, the prevalence of possible sarcopenia in urban area was 31.1% (95% CI 28.8, 33.4), whereas the rural areas accounted for 41.0% (95% CI 39.5, 42.4) (p<0.001).

**Table 1:** Prevalence and 95% CIs of possible sarcopenia by gender and living areas.

	Overall, %	Rural area, %	Urban area, %
Both genders	38.5(37.3, 39.7)	41.0(39.5, 42.4)	31.1(28.8, 33.4)
Men	36.3(34.6, 38.0)	38.4(36.4, 40.4)	29.8(26.5, 33.0)
Women	40.7(38.9, 42.4)	43.5(41.5, 45.5)	32.4(29.1, 35.6)

Mean handgrip strength was 18.10 (SD: 5.83) kg, 24.73 (SD: 4.47) kg in women vs 26.46 (SD: 7.02) kg, 36.29 (SD: 5.22) kg in men with possible sarcopenia and without sarcopenia, respectively. 5-time chair stand test was 13.01 (SD: 4.69) s and 8.50 (SD: 1.88) s in participants with possible sarcopenia and without sarcopenia, respectively. Compared with the participants without sarcopenia, those with possible sarcopenia were more likely to have hypertension, diabetes or high blood sugar, chronic lung diseases, heart disease, stroke, psychiatric disease, memory-related disease, arthritis and asthma (*p*<0.05), as is shown in Table 2. The possible sarcopenic participants were more likely to fall and fracture (*p*<0.001). The participants with possible sarcopenia had lower ASM, ASM/Ht^2^, gait speed (GS) and hemoglobin compared with those without sarcopenia (*p*<0.001). Inversely, the participants with possible sarcopenia had higher level of CRP, than those without sarcopenia (*p*<0.001) (Table 2).

**Table 2:** Characteristics of participants with or without possible sarcopenia.

Characteristic	No sarcopenia n = 3796 (61.5%)	Possible sarcopenia n = 2376 (38.5%)	*p*
Age (years) (mean ±SD)	66.49±5.35	70.74±7.17	<0.001
Age category, n (%)			
60–69	2826(74.4)	1160(48.8)	
70–79	883(23.3)	905(38.1)	
≥80	87(2.3)	311(13.1)	
Gender, n (%)			0.001
Male	1955(51.5)	1115(46.9)	
Female	1841(48.5)	1261(53.1)	
Area, n (%)			<0.001
Urban area	1061(28.0)	479(20.2)	
Rural area	2735(72.0)	1897(79.8)	
BMI (kg/m^2^)	24.36±16.22	24.05±23.20	0.537
BMI category, n (%)			
BMI<18.5	210 (5.5)	275 (11.6)	
18.5≤BMI<24	1920 (50.6)	1203 (50.6)	
BMI≥24	1666 (43.9)	898 (37.8)	
Smoking, n (%)	1811 (47.7)	1109 (46.7)	0.432
Alcohol consumption, n (%)	1382 (36.4)	661 (27.8)	<0.001
Comorbidities, n (%)			
Hypertension	1501(39.5)	1143(48.1)	<0.001
Dyslipidemia	704(18.5)	464(19.5)	0.350
Diabetes or high blood sugar	431(11.4)	323(13.6)	0.009
Cancer	74(1.9)	48(2.0)	0.851
Chronic lung diseases	671(17.7)	584(24.6)	<0.001
Liver disease	241(6.3)	148(6.2)	0.872
Heart disease	769(20.3)	602(25.3)	<0.001
Stroke	127(3.3)	138 (5.8)	<0.001
Kidney disease	390 (10.3)	258 (10.9)	0.468
Digestive disease	1153 (30.4)	769(32.4)	0.10
Psychiatric disease	117 (3.1)	110 (4.6)	0.002
Memory-related disease	117 (3.1)	124 (5.2)	<0.001
Arthritis	1686 (44.4)	1256 (52.9)	<0.001
Asthma	255 (6.7)	261 (11.0)	<0.001
Fall, n (%)	665(17.5)	567(23.9)	<0.001
Fracture, n(%)(n = 6165)	65(1.7)	75(3.2)	<0.001
ASM (mean±SD)	17.13±5.32	15.95±6.42	<0.001
Male	20.35±3.31	19.29±6.68	<0.001
Female	13.72±4.90	12.99±4.44	<0.001
ASM / Ht^2^ (kg/m^2^) (mean±SD)	6.82±2.38	6.56±3.28	<0.001
Male	7.60±1.21	7.40±2.25	0.001
Female	5.99±2.96	5.82±3.83	0.172
Gait speed(m/s) (mean±SD)	0.83±0.21	0.69±0.21	<0.001
Haematological Parameters (mean±SD)			
Hemoglobin, g/dl (n = 6092)	13.72±1.82	13.36±1.86	<0.001
C-Reactive Protein, mg/l (n = 6142)	2.60±5.11	3.56±8.05	<0.001

Results are presented as mean ± SD, or n (%). BMI, body mass index; ASM, appendicular skeletal muscle. P < 0.05 was considered to be statistically significant.

### The prevalence of participants with sarcopenia

As shown in S1 Table, sarcopenia had an overall prevalence of 18.6% (95% CI 17.7, 19.6). The prevalence of sarcopenia was 18.4% (95% CI 17.0, 19.7) among males and 18.9% (95% CI 17.5, 20.3) among females (*p* = 0.578). As shown in S1 Table and Fig 3, the prevalence of participants with sarcopenia was 9.4% in urban area (95% CI 8.0, 10.9), and was 21.7% (95% CI 20.5, 22.9) in rural area (*p*<0.001). The participants with sarcopenia were older compared with those without sarcopenia (mean age 72.39 years vs. 67.15 years, respectively, *p*<0.001). As shown in Fig 2, sarcopenia also became more common with age: 11.5%, 27.2%, and 52.0% in the 60–69, 70–79, and ≥80 years groups, respectively.

### The prevalence of participants with severe sarcopenia

As shown in S2 Table, the overall prevalence of severe sarcopenia was 8.0% (95% CI 7.3, 8.6). As shown in S2 Table and Fig 3, subjects in rural area (9.2%, 95% CI 8.4, 10.1) had a higher prevalence of severe sarcopenia than those in urban area (4.1%, 95% CI 3.1, 5.1) (*p* < 0.001). The prevalence of severe sarcopenia was 8.4% (95% CI 7.4, 9.4) in men and 7.5% (95% CI 6.6, 8.4) in women. The participants with severe sarcopenia were older compared with those without sarcopenia (mean age 75.01 vs. 67.53 years, respectively, *p*<0.001). As shown in Fig 2, the prevalence of severe sarcopenia increased with age: 3.3%, 12.3%, and 35.2% in the 60–69, 70–79, and ≥80 age groups, respectively.

### Relationship between appendicular skeletal muscle and handgrip strength

**Fig 4:**
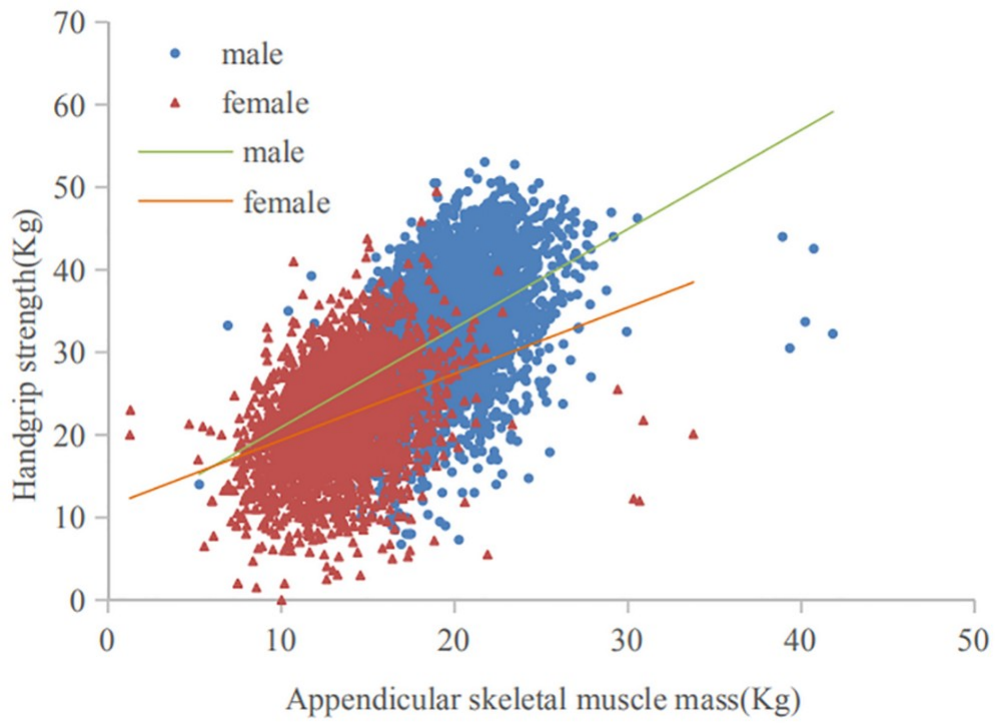
Correlation of appendicular skeletal muscle mass and handgrip strength for males and females. For males, regression equation: y = 1.204*x+8.823, adjusted R^2^ = 0.197, *p*<0.001; For females, regression equation: y = 0.820*x+11.119. adjusted R^2^ = 0.127, *p*<0.001.

The correlations between appendicular skeletal muscle and handgrip strength for males and females were shown in Fig 4. Handgrip strength was significantly positively correlated with appendicular muscle mass in males (correlation coefficients: 0.444, *p* <0.001). Handgrip strength was also significantly positively correlated with appendicular muscle mass in females (correlation coefficients: 0.356, *p* <0.001). Handgrip strength increased with appendicular muscle mass in both males and females (*p*<0.001).

### Associated factors for possible sarcopenia

Factors associated with possible sarcopenia were selected from the univariate analysis (Table 2). Any variables with *p*≤0.10 were entered into the multivariate logistic regression model. The H-L test of multivariate logistic regression gave *p* = 0.664.

As is shown in Table 3, possible sarcopenia became more common with age [odds ratio (OR) 1.09; 95% confidence interval (CI) 1.08–1.10]. The prevalence of possible sarcopenia in rural region was higher than urban region (OR 1.47; 95% CI 1.29–1.69). Having history of hypertension (OR 1.22; 95% CI 1.09–1.38), chronic lung diseases (OR 1.23, 95% CI 1.07–1.42), heart disease (OR 1.15; 95% CI 1.00–1.33), emotional or psychiatric problems (OR 1.48; 95% CI = 1.10–1.99), arthritis (OR 1.21; 95% CI 1.07–1.35) were associated with a higher risk of possible sarcopenia. Older adults who had fallen were more likely to have possible sarcopenia (OR 1.24; 95%CI 1.07–1.42). The high concentration of CRP was related to high risk of possible sarcopenia (OR 1.02; 95% CI 1.01–1.03). Conversely, alcohol consumption (OR 0.85; 95% CI 0.75–0.97), gait speed (OR 0.09; 95% CI 0.07–0.12), and hemoglobin (OR 0.97; 95% CI 0.94–1.00) were negatively associated with the risk of possible sarcopenia. However, the associations between possible sarcopenia with alcohol consumption, heart disease, psychiatric disease and high levels of hemoglobin were not significant after correction for multiple comparisons (Table 3).

**Table 3:** Multivariate logistic regression of the potentially associated risk factors for possible sarcopenia.

Variables	OR (95%CI)	*p*-Value
Age	1.09(1.08–1.10)	**<0.001**
Area		
Urban area	1(reference)	
Rural area	1.47(1.29–1.69)	**<0.001**
Alcohol consumption	0.85(0.75–0.97)	0.013
Gait speed(m/s)	0.09(0.07–0.12)	**<0.001**
Hypertension	1.22(1.09–1.38)	**0.001**
Chronic lung diseases	1.23(1.07–1.42)	**0.004**
Heart disease	1.15(1.00–1.33)	0.048
Psychiatric disease	1.48(1.10–1.99)	0.010
Arthritis	1.21(1.07–1.35)	**0.002**
Fall	1.24(1.07–1.42)	**0.003**
Hemoglobin, g/dl	0.97(0.94–1.00)	0.034
C-Reactive Protein, mg/l	1.02(1.01–1.03)	**0.001**

OR: Odds ratio; CI: confidence interval. N = 6061. Goodness-of-fit: H-L Chi^2^ (8) = 5.853, *p* = 0.664. Bold p-values: Indicates *p*-values that remain significant after Bonferroni comparisons (*p*<0.0042).

We also performed subgroup analysis according to the rural area and urban area. There were some differences regarding the associated factors of sarcopenia in different districts. We found age, gait speed, arthritis and fall were common associated factors for possible sarcopenia regardless of urban or rural residence(*p***<**0.05). Having chronic lung diseases (OR 1.42; 95% CI 1.06–1.90) and psychiatric disease (OR 2.21; 95% CI 1.23–3.96) were associated with a higher risk of possible sarcopenia among urban populations. On the contrary, alcohol consumption (OR 0.71; 95% CI 0.54–0.92) was negatively associated with the risk of possible sarcopenia in urban areas. The risk for possible sarcopenia was greater among individuals with hypertension(OR1.29; 95% CI 1.13–1.48), heart disease (OR 1.24; 95% CI 1.05–1.47) or high concentration of CRP(OR1.02; 95%CI 1.01–1.03) in rural populations, whereas hemoglobin (OR 0.96; 95% CI 0.92–0.99) was negatively associated with the risk of possible sarcopenia in rural populations. After correction for multiple comparisons, only age and gait speed were significantly associated factors for possible sarcopenia regardless of urban or rural residence(*p***<**0.001). The risk for possible sarcopenia was significantly greater among individuals with hypertension, or high concentration of CRP in rural populations(*p***<**0.001) (Table 4).

**Table 4:** Logistic regression models for possible sarcopenia in urban and rural populations.

Variables	Urban	*p*-Value	Rural	*p*-Value
	OR (95%CI)		OR (95%CI)	
Age	1.09(1.07–1.11)	**<0.001**	1.09(1.08–1.10)	**<0.001**
Alcohol consumption	0.71(0.54–0.92)	0.011	0.90(0.78–1.04)	0.138
Gait speed(m/s)	0.11(0.06–0.20)	**<0.001**	0.08(0.06–0.12)	**<0.001**
Hypertension	1.02(0.80–1.30)	0.895	1.29(1.13–1.48)	**<0.001**
Chronic lung diseases	1.42(1.06–1.90)	0.021	1.18(1.00–1.38)	0.051
Heart disease	0.94(0.72–1.23)	0.667	1.24(1.05–1.47)	0.010
Psychiatric disease	2.21(1.23–3.96)	0.008	1.27(0.90–1.78)	0.179
Arthritis	1.29(1.01–1.64)	0.046	1.18(1.03–1.35)	0.014
Fall	1.49(1.09–2.04)	0.012	1.18(1.00–1.38)	0.046
Hemoglobin, g/dl	1.01(0.94–1.09)	0.770	0.96(0.92–0.99)	0.012
C-Reactive Protein, mg/l	1.00(0.98–1.02)	0.909	1.02(1.01–1.03)	**<0.001**

OR: Odds ratio; CI: confidence interval. Bold *p*-values: Indicates p-values that remain significant after Bonferroni comparisons.

## Discussion

To our knowledge, this study is the first study to estimate the prevalence of sarcopenia and explore the associated factors with possible sarcopenia using the national survey data of CHARLS among older Chinese population aged ≥60 years. According to the updated diagnostic criteria of AWGS 2019, we found that the overall prevalence of possible sarcopenia, sarcopenia and severe sarcopenia in the study population, was 38.5%, 18.6%, and 8.0%, respectively and increased with age. We identified a range of factors associated with sarcopenia prevalence. Some of associated factors have for the first time been shown to be associated with sarcopenia in Chinese population.

The prevalence of sarcopenia is different across studies due to different study populations (e.g., age, gender) and the adoption of different diagnosis criteria [20, 21]. According to a systematic review, the prevalence of sarcopenia among adults aged 50 years and older ranged from 1% to 29% in community dwelling populations [20]. A Chinese study involving 944 community dwelling older adults aged ≥60 years showed that the prevalence of AWGS 2014-defined sarcopenia was approximately 10.4% [22]. However, another study including 887 community-dwelling elderly Chinese adults reported the sarcopenia prevalence rate was 9.8% [23]. The AWGS 2019- defined sarcopenia was new. Therefore, to our knowledge, few relevant studies have been published so far using the new definition. We found only one study about sarcopenia in Korean community-dwelling adults aged 70 years and older applying the recently updated AWGS 2019 diagnostic algorithm. The authors reported that the prevalence of sarcopenia was 22.8% [24]. However, the prevalence of sarcopenia was 18.6% in our study according to the latest diagnostic criteria of AWGS 2019. The differences in the prevalence may be related to different study populations and measurement methods used. In addition, the Korean subjects were older than those in our study (75.9 ±3.9 years Vs 68.13±6.46 years, respectively), which might also explain why the prevalence of sarcopenia was higher in the elderly population of Korea than ours.

It is well known that sarcopenia is an age-related loss of muscle mass, has been described as a decline in muscle mass and muscle strength associated with aging [25]. This is consistent with our study. We found the prevalence of possible sarcopenia obviously increased with age (OR 1.09; 95% CI 1.08–1.10). Our study indicated that drinking might be a protective factor for possible sarcopenia (OR 0.85; 95% CI 0.75–0.97). However, the association between possible sarcopenia with alcohol consumption was not significant after Bonferroni correction. A previous study showed that lifestyle habits such as chronic alcohol consumption might result in loss of muscle mass and strength in older adults indicating that they could be risk factors for sarcopenia [26]. But the benefits of alcohol consumption are also recognized. In common with our study, a meta-analysis reported alcohol consumption was not a risk factor for the sarcopenia, even more, alcohol consumption could have protective character against sarcopenia [27]. Therefore, further studies are needed to establish the link between alcohol consumption and sarcopenia. In common with previous studies [23], we found that rural region had a higher prevalence rate of possible sarcopenia than urban region (OR 1.47; 95% CI 1.29–1.69). It may be explained by the fact that the socioeconomic development in urban region may contribute to better public health. However, many rural elders may suffer from malnutrition or were at risk for malnutrition, which may cause lower muscle mass and muscle strength. We also performed subgroup analysis according to the rural area and urban area. There were some differences regarding the associated factors of sarcopenia in different districts. Therefore, different strategies should be taken in rural and urban areas to prevent sarcopenia.

Plenty of evidence showed that age-related chronic low-grade inflammation could be a vital contributor of sarcopenia [28]. Previous studies showed that high levels of proinflammatory cytokines were associated with the loss of muscle mass and poor physical function in older adults [29, 30]. We also found CRP among older Chinese population with possible sarcopenia was significantly higher than those without sarcopenia. Previous studies have demonstrated that the prevalence of sarcopenia increased in people with COPD [31], cardiovascular diseases [32]. In our study, we found that chronic lung diseases (OR 1.23, 95% CI 1.07–1.42), hypertension (OR 1.22; 95% CI 1.09–1.38), heart disease (OR 1.15; 95% CI 1.00–1.33), arthritis (OR 1.21; 95%CI 1.07–1.35) were associated with an increased risk for possible sarcopenia. However, the association between possible sarcopenia with heart disease was not significant after correction for multiple comparisons. Systemic inflammation could be an important contributing factor of sarcopenia in the COPD population [33]. Lack of physical exercise is relevant to the loss of muscle function at early stage in COPD patients [33]. Chronic inflammation and the production of cytokine is a normal phenomenon associated with aging and is the predominant risk factor for ageing-related chronic diseases, such as hypertension [34], cardiovascular diseases [35]. Patients with rheumatoid arthritis are especially vulnerable to developing sarcopenia in view of the underlying pro-inflammatory state and the reduction in muscle use because of physical inactivity and pain. Patients with rheumatoid arthritis lose two-three times as much muscle mass and function [36]. A previous systematic review reported that cognitive impairment (e.g., dementia) was associated with sarcopenia [37]. However, we did not find the association between memory-related disease (e.g., dementia) and sarcopenia in older Chinese adults. Interestingly, we found psychiatric disease was associated with an increased risk of possible sarcopenia with OR of 1.48 (95% CI1.10–1.99). Nevertheless, the association was no longer significant after Bonferroni correction. A meta-analysis indicated that some proinflammatory cytokines, including tumor necrosis factor-alpha and interleukin-6, were connected with depressive symptoms [38]. Inflammation has also been shown to be an important contributor of sarcopenia [29]. Therefore, inflammation might be one of common etiopathogenesis both sarcopenia and psychiatric. A previous study reported associations between inflammatory cytokine and physical function in the older adults with multiple comorbidities [39]. The researchers demonstrated that the increase of CRP and IL-6 levels is related to poorer physical function in older adults with various comorbidities [29]. Based on the above, for the potential biological mechanisms, systemic inflammation caused by inflammatory cytokines (e.g., CRP) may be one of contributing factors to sarcopenia and related comorbidities such as COPD, hypertension, arthritis, and so on. The other potential mechanism may be explained by the fact that those having a history of chronic diseases are predisposed to decrease the levels of physical activity which further lead to disuse-atrophy of muscle.

The previous study has indicated that sarcopenia was significantly associated with fall in elderly population [40]. We got the similar result in the present study with an increased risk of possible sarcopenia with OR of 1.24, and 95% CI of 1.07–1.42. A systematic review showed that there were different directions of causal pathways between falls and sarcopenia. In other words, sarcopenia as a cause for falls, and falls as a cause for sarcopenia [41]. Furthermore, we found lower gait speed exists in the old adults with sarcopenia. The existence of sarcopenia is more likely to lead to adverse consequences for older adults (e.g., falls, lower gait speed). In addition, we found hemoglobin (OR 0.97; 95% CI 0.94–1.00) was associated with a lower likelihood of possible sarcopenia. However, the association between possible sarcopenia with hemoglobin was not significant after correction for multiple comparisons.

Our study has several strengths. First, it was conducted in a nationwide representative sample. The findings can be generalized to all older Chinese adults. Second, we firstly estimated the prevalence of three categories of sarcopenia according to the updated diagnostic criteria of AWGS 2019. Third, besides sarcopenia prevalence, we reported a number of factors associated with possible sarcopenia. Such information will be helpful for health planning and prevention of sarcopenia in China. Fourth, we found rural elders were more vulnerable to sarcopenia than urban elders among older Chinese population. More attention should be paid to rural populations in future studies. Additionally, we found chronic inflammation caused by inflammatory cytokines (e.g., CRP) might be one of the contributing factors for sarcopenia and related comorbidities. Finally, our findings will provide clues for future studies.

There are still limitations in this study. First, for the definiation of sarcopenia, we used an anthropometric equation to estimate the muscle mass, which has previously been validated in Chinese individuals [12, 42, 43], instead of Dual X-ray absorpometry (DXA) or Bioelectrical impedance analysis (BIA) recommended by the AWGS 2019 [3]. However, in the previous study by Furushima et al., estimation formulas for appendicular muscle mass using anthropometric values has been reported, and its validity has been confirmed [44]. Besides, it has been demonstrated that the use of anthropometric equations to estimate the muscle mass combined with the handgrip strength and gait speed may comprise a cost-effective alternative to DXA to improve the diagnosis of sarcopenia [45], given the risk of X-ray exposure of DXA and high cost of measurements. For BIA, the estimates of muscle mass vary when different instrument brands and reference populations are used [1]. Furthermore, we demonstrated the estimated appendicular skeletal muscle mass was significantly positively correlated with handgrip strength in both males and females which also proved that the estimation of muscle mass was valuable, to some extent. Second, we used a 2.5-m walk to test gait speed rather than 6-m walk recommended by the AWGS 2019. However, we found the average gait speed of the population (68.13±6.46 years) was 0.68±0.19 m/s, 0.80±0.22 m/s in the participants with sarcopenia and without sarcopenia, respectively, which was consistent with the result of a previous Chinese study. Langli Gao, et al. showed the average gait speed of community-dwelling older Chinese adults (70.6±6.7 years) was 0.6±0.2 m/s, 0.8±0.2 m/s in the participants with sarcopenia and without sarcopenia, respectively, in a 20-m walk [23]. In addition, a systematic review included 48 studies assessing gait speed in the older found that the “distance walked during the gait speed test did not influence the recorded gait speed” [46]. Therefore, 2.5-m walk might be appropriate for assessing the gait speed of older Chinese adults. Third, we didn’t include the screening of case finding and measure of SPPB in our study. Thus, the estimates may have some degree of bias. Fourth, though we considered as many relevant factors as possible in the present study, unmeasured factors (e.g. nutritional status) may be important factors associated with possible sarcopenia. Fifth, CHARLS has amounts of missing or incomplete data. Incomplete datasets may lead to potential biases. However, we used these data to analyze the associated factors with possible sarcopenia, some findings were consistent with previous studies.

## Conclusions

In conclusion, we found the prevalence and a range of factors associated with sarcopenia among older Chinese population according to the diagnostic criteria of AWGS 2019. These findings of our study are important as they will contribute to increase awareness of sarcopenia and treat patients at risk for sarcopenia to take actions that will promote early detection and treatment in Chinese population. Additionally, large population-based perspective surveys are required to establish ethnic-specific references and cut-off values for Chinese people.

## Supporting information

S1 Table Prevalence and 95% CIs of sarcopenia by gender and living areas. (DOCX)

S2 Table Prevalence and 95% CIs of severe sarcopenia by gender and living areas. (DOCX)
