# Supplementary material for: Sarcopenia prevalence and associated factors among older Chinese population: Findings from the China Health and Retirement Longitudinal Study
Source: PLoS One. 2021 Mar 4;16(3):e0247617. doi: 10.1371/journal.pone.0247617 (PMC7932529; doi:10.1371/journal.pone.0247617)
Supplement: S1 Table — (DOCX) [file pone.0247617.s001.docx]

|  | Overall,% | Rural area,% | Urban area, % |
| --- | --- | --- | --- |
| Both genders | 18.6(17.7, 19.6) | 21.7(20.5, 22.9) | 9.4(8.0, 10.9) |
| Men | 18.4(17.0, 19.7) | 21.4(19.8, 23.1) | 8.8(6.8, 10.9) |
| Women | 18.9(17.5, 20.3) | 22.0(20.3, 23.7) | 9.9(7.9,12.0) |
