# Supplementary material for: Sarcopenia prevalence and associated factors among older Chinese population: Findings from the China Health and Retirement Longitudinal Study
Source: PLoS One. 2021 Mar 4;16(3):e0247617. doi: 10.1371/journal.pone.0247617 (PMC7932529; doi:10.1371/journal.pone.0247617)
Supplement: S2 Table — (DOCX) [file pone.0247617.s002.docx]

|  | Overall,% | Rural area,% | Urban area, % |
| --- | --- | --- | --- |
| Both genders | 8.0(7.3, 8.6) | 9.2(8.4, 10.1) | 4.1(3.1, 5.1) |
| Men | 8.4(7.4, 9.4) | 9.8(8.6, 11.0) | 4.0(2.6, 5.4) |
| Women | 7.5(6.6, 8.4) | 8.7(7.5, 9.8) | 4.2(2.8, 5.5) |
